# Approximate Genome-Based Kernel Models for Large Data Sets Including Main Effects and Interactions

**DOI:** 10.3389/fgene.2020.567757

**Published:** 2020-10-15

**Authors:** Jaime Cuevas, Osval A. Montesinos-López, J. W. R. Martini, Paulino Pérez-Rodríguez, Morten Lillemo, Jose Crossa

**Affiliations:** ^1^Universidad de Quintana Roo, Chetumal, Mexico; ^2^Facultad de Telemática, Universidad de Colima, Colima, Mexico; ^3^International Maize and Wheat Improvement Center (CIMMYT), Texcoco, Mexico; ^4^Colegio de Postgraduados, Texcoco, Mexico; ^5^Department of Plant Sciences (IPV), Norwegian University of Life Sciences (NMBU), Ås, Norway

**Keywords:** genomic-enabled prediction, approximate kernels, computing time, genotype × environment interaction, large data sets

## Abstract

The rapid development of molecular markers and sequencing technologies has made it possible to use genomic prediction (GP) and selection (GS) in animal and plant breeding. However, when the number of observations (*n*) is large (thousands or millions), computational difficulties when handling these large genomic kernel relationship matrices (inverting and decomposing) increase exponentially. This problem increases when genomic × environment interaction and multi-trait kernels are included in the model. In this research we propose selecting a small number of lines *m*(*m* < *n*) for constructing an approximate kernel of lower rank than the original and thus exponentially decreasing the required computing time. First, we describe the full genomic method for single environment (FGSE) with a covariance matrix (kernel) including all *n* lines. Second, we select *m* lines and approximate the original kernel for the single environment model (APSE). Similarly, but including main effects and G × E, we explain a full genomic method with genotype × environment model (FGGE), and including *m* lines, we approximated the kernel method with G × E (APGE). We applied the proposed method to two different wheat data sets of different sizes (*n*) using the standard linear kernel Genomic Best Linear Unbiased Predictor (GBLUP) and also using eigen value decomposition. In both data sets, we compared the prediction performance and computing time for FGSE versus APSE; we also compared FGGE versus APGE. Results showed a competitive prediction performance of the approximated methods with a significant reduction in computing time. Genomic prediction accuracy depends on the decay of the eigenvalues (amount of variance information loss) of the original kernel as well as on the size of the selected lines *m*.

## Introduction

The rapid development of molecular markers and sequencing technologies has made it possible to use genomic prediction (GP) and selection (GS) in animal and plant breeding (Meuwissen et al., 2001), and practical evidence in plant and animal breeding data has shown that GS provides important prediction accuracy for GS-assisted breeding (Meuwissen et al., 2001; Crossa et al., 2010, 2011; de los Campos et al., 2010; Pérez-Rodríguez et al., 2012).

Additive genetic effects can be predicted directly from marker effects by Ridge Regression best linear unbiased prediction (rrBLUP) (Endelman, 2011) and/or by employing Bayesian inference (Meuwissen et al., 2001), and/or developing the genomic relationship linear kernel matrix (***G***) to fit the GBLUP (VanRaden, 2008). The GBLUP has the advantage of mitigating the high dimension problem and is flexible enough to be extended to more complex situations like incorporating genotype × environment interactions (GE) or studying multi-traits and multi-environments with multi-kernel methods (Jarquín et al., 2014; Lopez-Cruz et al., 2015). The ***G*** of the GBLUP method is a linear kernel (***K***), since it models the additive lineal relationship between lines.

Departures from linearity can be assessed by semi-parametric approaches, such as mixed models with non-additive covariance structure defined in the Reproducing Kernel Hilbert Space (RKHS) framework or more complicated prediction methods such as neural networks (Gianola et al., 2006; Gianola and van Kaam, 2008; de los Campos et al., 2010; González-Camacho et al., 2012; Pérez-Rodríguez et al., 2012). Gianola et al. (2006, 2014) suggested using RKHS regression for semi-parametric, genomic-enabled prediction and pointed out that non-parametric methods such as kernel regression are necessary to reduce the dimension of the parametric space, and to be able to capture complex cryptic interaction among markers. The most commonly used nonlinear kernels in the Reproducing Kernel Hilbert Space (RKHS) (Gianola et al., 2006, 2014) is the Gaussian kernel (GK) that can be expressed dually as a marker effect and interaction effect model (epistasis) (Martini et al., 2020). The Gaussian kernel (GK) for estimating genetic values captures more complex relationships between markers using the Euclidean distance as the dissimilarity between lines based on molecular markers and estimating a bandwidth parameter (*h*) (de los Campos et al., 2010). Thus, a Gaussian kernel function is $K_{h}\left(x_{i},x_{i^{\prime }}\right)=\exp\;\left(-hd_{ii^{\prime }}^{2}/q\right)$, where $x_{i},x_{i^{\prime }}$ are the marker vectors for the *i*th and *i’*th individuals, and *q* is a scale factor that can be fixed by the user with the idea of reducing the value of *h*; in general it is a percentile of the squared Euclidean distance $d_{ii^{\prime }}^{2}$ for example, the fifth percentile of the squared Euclidean distance $d_{ii^{\prime }}^{2}$ (Pérez-Rodríguez et al., 2012), or the 50 percentile used by Crossa et al. (2010).

Standard GS models were extended to multi-environments by assessing genomic × environment interaction (GE) (Burgueño et al., 2012). Jarquín et al. (2014) proposed an extension of the GBLUP that is a type of random effects model where the main effects of markers and environmental covariates (ECs), as well as the interactions between markers and ECs, are introduced using covariance structures that are functions of marker genotypes and ECs. The proposed approach can be interpreted as a random effects model on all the markers, all the ECs, and all the interactions between markers and ECs using a multiplicative operator. Lopez-Cruz et al. (2015) proposed a marker × environment interaction model where the marker effects and genomic values are partitioned into components that are stable across environments (main effects) and others that are environment-specific (interactions); this interaction model is useful when selecting for stability and for adaptation to targeted environments. Consistently, genomic prediction accuracy substantially increased when incorporating GE and marker × environment interaction (Crossa et al., 2017). The marker × environment interaction model has some advantages over previous models; it is easy to implement in standard software for GS like the BGLR (de los Campos and Pérez-Rodríguez, 2018) or the BGGE (Granato et al., 2018), and it can also be implemented with any priors commonly used in GS, including not only shrinkage methods (e.g., GBLUP), but also variable selection methods (that could not be directly implemented under the reaction norm model) (Crossa et al., 2016).

Cuevas et al. (2016) applied the marker × environment interaction GS model of Lopez-Cruz et al. (2015) but modeled not only through the standard linear kernel (GBLUP) but also through a nonlinear Gaussian kernel similar to that used in the Reproducing Kernel Hilbert Space with Kernel Averaging (RKHS KA) (de los Campos et al., 2010) and a Gaussian kernel with the bandwidth estimated through an empirical Bayesian method (Pérez-Elizalde et al., 2015). The methods proposed by Cuevas et al. (2016) were used to perform single-environment analyses and extended to account for GE interaction in wheat and maize data sets. Cuevas et al. (2016) concluded that the higher prediction accuracy of the Gaussian kernel models with the GE model is due to more flexible kernels that allow accounting for small, more complex marker main effects and marker-specific interaction effects.

In the Ridge Regression rrBLUP (Kang et al., 2008; Endelman, 2011), the reduced dimensionality advantages of eigen decomposition were used to estimate the variance components by means of maximum likelihood and/or restrictive maximum likelihood (REML) to compute the genomic-enabled predictions. Pérez-Elizalde et al. (2015) also used eigen decomposition with the marginal maximum likelihood to estimate the genetic and the residual variance components. Pérez-Rodríguez and de los Campos (2014) developed a very useful and efficient statistical software for Bayesian Generalized Linear Regression (BGLR) based on Monte Carlo Markov Chain (MCMC). Granato et al. (2018) also used the spectral decomposition with covariance matrices of exact rank when employing a Bayesian approach.

However, in GP, not only is the number of markers large, but also the number of individuals could be high, thus making the complete kernel matrix difficult to manipulate, and computationally very intensive. This significant increase in the number of observations (individuals) is common when the genomic-enabled prediction model includes genotype × environment interaction (GE) with different and large numbers of lines in each environment (or year) (Jarquín et al., 2014). In these models, the covariance matrices of the main effects and interactions usually have ranks smaller than the number of observations (lines). In these cases, exact low rank matrices are commonly employed, as in rrBLUP (Kang et al., 2008; Endelman, 2011), as well as the Bayesian Genomic Genotype × Environment interaction (BGGE) software of Granato et al. (2018); however, eigen decomposition also has a high computational cost when both the number of observations and markers is large.

An alternative way to deal with large data sets is to use methods such as approximate kernels with the objective of reducing the computational processing time without affecting the genomic prediction accuracy very much. This methodology is commonly used in the framework of machine learning and in the Gaussian process (Rasmussen and Williams, 2006), where the main problem is the large number of observations (e.g., several thousands), whereas the number of covariates (markers) is not that large.

Wang et al. (2015) commented that the method of approximate kernels could be useful for GP when the number of observations is large. The application of GBLUP or GK is practically intractable for deriving the eigen decomposition of large *n* because of the time scale and the storage capacity. In animal genomic selection, Misztal (2016) proposed a method to approximate a linear kernel relationship matrix using a small size of the original large training population with the objective of facilitating the inversion of the genomic matrix and being able to employ a single-step method when predicting the performance of a large number of animals.

Lately, a number of new ideas and algorithms have addressed the problem of determining input that is relevant for predicting the output, that is, it is possible to develop an efficient predictive model that does use all the large *n* observations, but approximates the kernels with a low rank. The method of approximate kernels seems to achieve this objective by proposing a simple input that originally had a kernel matrix ***K***_*n*,*n*_ of order *n* × *n* from where a smaller sub-matrix is selected, ***K***_*m*,*m*_ of order *m* × *m* with the restriction that *m* < *n*, with the objective of finding an approximate matrix ***Q*** of rank *m*, smaller than the rank of the original matrix (Seeger et al., 2003). That is,

$$K\approx Q=K_{n,m}K_{m,m}^{-1}K_{n,m}^{\prime }$$

where ***K***_*m*,*m*_ is a sub-matrix of the initial ***K*** = ***K***_*n*,*n*_ and can be constructed with *m* selected lines with *p* markers where ***K***_*n*,*m*_ is a sub-matrix of ***K*** with the relation between the total *n* lines and the *m* selected ones. Therefore, ***Q*** is an approximation of ***K***, but of smaller rank (*m*), so that computational time is significantly saved when performing the required spectral decomposition or/and inversion. Based on this approximation, a large number of methods have been proposed, such as the projected process of Seeger et al. (2003), which assumes *a priori* that the random effects have a covariance matrix of $\sigma_{u}^{2}$ ***Q***. Also, Snelson and Ghahramani (2006) proposed correcting the diagonal of ***Q*** in order to propose a method of pseudo points. Furthermore, a similar approximate method was proposed and implemented by Misztal et al. (2014) and Misztal (2016), who employed recursive methods from the joint distribution of the random genetic effects when testing a large amount of animal production. Titsias (2009) proposed a variational perspective that maximizes the lower bound of the exact marginal likelihood by incorporating, as a penalized element, the trace of the differences of matrices ***K***, ***Q***. Hensman et al. (2013) presented a stochastic variational method and found a lower limit than the one reported by Titsias (2009).

In general, approximate kernel methods could be useful when the size of the training set is large and the construction of the matrices and their manipulations in terms of storage, inversion and decomposition are highly computing intensive and practically prohibitive (Rasmussen and Williams, 2006). On the other hand, the main concern is how the quality of the approximations would be in terms of genomic-enabled prediction. According to Wang et al. (2015), the eigenvalue decomposition of these full matrices decays rapidly, thus favoring the use of these approximations (Rasmussen and Williams, 2006). Based on the previous difficulties in assessing efficient computer-scale time of genomic problems when the number of observations is large, we have adopted an approximate kernel method for large data using a Bayesian approach to be used in genomic-enabled prediction R packages like BGLR (Pérez-Rodríguez and de los Campos, 2014). To test our proposed approximate method, we used two wheat data sets, one of which is relatively small and the other very large. We compared the performance of the proposed approximate kernel versus the full kernel based on the genomic-enabled prediction accuracy, which in turn was measured based on the correlations between the observed and predictive values, the mean squared error and the estimation of the magnitude of the residual error. This method is valid for any kind of kernel; however, in this study we used it only with linear kernels.

## Materials and Methods

### Statistical Models and Methods

We named the conventional GBLUP the “full genomic model” (FG) and the approximation model the “genomic sparse kernel approximation model” (AP). Depending on whether the model is for single-environment (SE) analyses or for GE analyses, FG is called FGSE and FGGE, respectively, and the AP method is called APSE and APGE.

### The Full Genomic Method Single-Environment Model (FGSE)

To facilitate the description of this model, we first explain the basic parametric genetic model (assuming the fixed effects have been already considered)

(1) $$y=\mu 1_{n}+X\beta +\varepsilon$$

where ***y*** is the vector of observations of the response variable of size *n* × 1, μ is the overall mean, ***X*** is the matrix of the *p* markers on the *n* lines associated with ***y***, and ***β*** is the vector of the *p* marker effects, which in the Bayesian framework are considered random effects with normal distribution $N\left(0,\sigma_{\beta }^{2}I_{n}\right)$. Finally, random vector ε has normal distribution $N\left(0,\sigma_{\varepsilon }^{2}I_{n}\right)$, where $\sigma_{\varepsilon }^{2}$ is the variance component of the random errors and ***I***_*n*_ is an identity matrix of order *n*×*n*.

The previous model can be represented as a GBLUP model

(2) $$y=\mu 1_{n}+u+\varepsilon$$

where ***u*** is the vector of random effects of size *n* × 1 with $N\left(0,\sigma_{u}^{2}K\right)$, $\sigma_{u}^{2}$ is a scaled parameter to be estimated and ***K*** is a known positive semidefinite matrix of order *n* × *n*, constructed based on molecular markers ***X*** of order *n* × *p*, where *p* denotes the number of markers such that $K=\frac{{\mathrm{XX}}^{\prime }}{p}$ is known as GBLUP (VanRaden, 2008; Lopez-Cruz et al., 2015). Note that there is no incidence matrix for ***u*** because ***K*** is constructed directly using the markers of model (1), which are in line with the response vector ***y***.

The eigenvalue decomposition of ***K*** is ***US***^1/2^ ***S***^1/2^ ***U***′, substituting **u** in model (2), is equivalent to

(3) $$y=\mu 1_{n}+\mathrm{Pf}+\varepsilon$$

where $f∼N\left(0,\sigma_{f}^{2}I_{r}\right)$, (where *r* is the rank of ***K***) and ***P*** = ***US***^1/2^. Note that models (1), (2) and (3) are equivalent. Models (1) and (3) can be fitted by the conventional Ridge regression model. The Ridge regression model can be computationally fitted very quickly, especially in situations where *r* < min (*n*, *p*), which is common in multi-environment and/or multi-trait models. It should be noted that only *r* effects can be summarized and projected for ***P*** to explain the *n* effects without any loss of precision with the available information.

### Genomic Approximate Kernel Methods for a Single-Environment Model (APSE)

First, the method considers ***K***, based on a smaller sub-matrix ***K***_*m*,*m*_(*m* < *n*) constructed with the markers of *m* lines. When the row vectors are linearly independent, the rank of *K*_*m,m*_ is *m*. Williams and Seeger (2001) showed that the Nyström approximation of the kernel is as follows:

$$K\approx Q=K_{n,m}K_{m,m}^{-1}K_{n,m}^{\prime }$$

where ***Q*** will have the rank of ***K***_*m*,*m*_, that is *m*. Note, however, that it is not necessary to compute and store the original matrix ***K***, only ***K***_*m*,*m*_ and ***K***_*n*,*m*_.

In this approximation, ***K***_*m*,*m*_ is constructed with *m* lines with all the *p* markers, that is, ***X***_*m*,*p*_. For the case of the GBLUP, $K_{m,m}=\frac{X_{m,p}X_{m,p}^{\prime }}{p}$ and $K_{n,m}=\frac{X_{n,p}X_{m,p}^{\prime }}{p}$ which captures the relationship of all *n* lines with the *m*. Note that in the construction of ***Q***, all the *p* markers and all the *n* lines are considered, but not all their relationships are accounted for; for example, relationships $K_{n-m,n-m}=\frac{X_{n-m,p}X_{n-m,p}^{\prime }}{p}$ are not considered (where *n-m* represents all the rest of the *m* lines). To try to explain this, we ordered the elements of matrix ***K*** per blocks, such that $K_{n,m}=\left[\begin{array}{cc} K_{m,m} & K_{m-n,m}\\ K_{n-m,m} & K_{n-m,n-m} \end{array}\right]$.

Rasmussen and Williams (2006) showed that ***Q***_*m*,*m*_ = ***K***_*m*,*m*_, ***Q***_*n*−*m*,*m*_ = ***K***_*n*−*m*,*m*_, ***Q***_*m*,*n*−*m*_ = ***K***_*m*,*n*−*m*_, and that the difference between ***K***_*n*−*m*,*n*−*m*_−***Q***_*n*−*m*,*n*−*m*_, that is, ***K***_*n*−*m*,*n*−*m*_−$K_{n-m,m}K_{m,m}^{-1}K_{m,n-m}$ is the Schur complement of ***K***_*m*,*m*_ on ***K***_*n*,*n*_. Then, because it is assumed that ***K***_*m*,*m*_ and ***K***_*n*,*n*_ are positive semidefinite, their Schur complement is also positive semidefinite: $Q_{n,m}=\left[\begin{array}{cc} K_{m,m} & K_{m-n,m}\\ K_{n-m,m} & Q_{n-m,n-m} \end{array}\right]$. Assuming the effects of ***u***_*n*−*m*_|***u***_*m*_ are conditional independent, Snelson and Ghahramani (2006) and Misztal et al. (2014) proposed substituting the diagonal of the differences of ***Q***_*n*−*m*,*n*−*m*_ with the diagonal of ***K***_*n*−*m*,*n*−*m*_.

In the method called Projected Process, Seeger et al. (2003) theoretically show that using all lines and considering the minimum Kullback-Leibler distance *KL*(*q*(***u***|*y*)||*p*(***u***|y)) justifies that matrix ***K*** in the prior distribution of ***u*** (of model 2) can be substituted for the ***Q*** approximations from Nyström (Titsias, 2009). That is, the random genetic vectors have a normal distribution $u∼N\left(0,\sigma_{u}^{2}Q\right)$, where $Q=K_{n,m}K_{m,m}^{-1}K_{n,m}^{\prime }$. More details are given in Csató and Opper (2002).

These adjustments in the distribution of the random effects ***u*** of model 2 can be done for genome-based prediction. It is common to estimate parameters $\sigma_{\varepsilon }^{2}$ and $\sigma_{u}^{2}$ of the model with the marginal likelihood by means of numerical methods and then predict them using the inversion lemma, which is fast when the model is for a single environment. However, the purpose of this study is to develop a methodology in order to jointly estimate and predict complex models such as genotype × environment interactions by making the eigen value decomposition transformation so that it allows us to use ridge regression or Bayesian ridge regression, which can be adjusted with diverse software. Furthermore, if matrix ***Q*** is directly used with model (2), the advantages (in terms of speed) of the approximate kernel would not apply. Therefore, similar to model (3), what we did is perform an eigen-decomposition of $K_{m,m}^{-1}=U_{m,m}S_{m,m}^{-1/2}{U^{\prime }}_{m,m}$, where $U_{m,m}$ are the eigenvectors of order *m*×*m* and ***S***_*m*,*m*_ is a diagonal matrix of order *m*×*m* with the eigenvalues ordered from largest to smallest. These values are substituted in ***Q*** resulting in $u_{n}∼N(0,\sigma_{u}^{2}K_{n,m}U_{m,m}S_{m,m}^{-1/2}\;S_{m,m}^{-1/2}{U^{\prime }}_{m,m}{K^{\prime }}_{n,m}$), and thus, due to the properties of the normal distribution, model (1) could be expressed as:

(4) $$y=\mu 1_{n}+\mathrm{Pf}+\varepsilon$$

Model (4) is similar to model (3), except that ***f*** is a vector of order *m*×1 with a normal distribution of the form $f∼N\left(0,\sigma_{f}^{2}I_{m}\right)$, where $P=K_{n,m}U_{m,m}S_{m,m}^{-1/2}$. This implies estimating only the *m* effects and expanding them in the *n* dimensional space in order to predict ***u***_*n*_ and explain ***y***_*n*_. Note that model (4) has a Ridge regression solution, and thus diverse software can be used.

In summary, the approximation described above consists of the following steps:

Step 1. Compute the matrix ***K***_*m*,*m*_ from *m* lines of the training set. The lines are randomly selected.
Step 2. Construct matrix ***K***_*n*,*m*_.
Step 3. Compute the eigenvalue decomposition of ***K***_*m*,*m*_.
Step 4. Compute matrix $P=K_{n,m}U_{m,m}S_{m,m}^{-1/2}$.
Step 5. Fit the model and make genomic-enabled predictions with Bayesian Ridge Regression or Ridge Regression.

### The Full Genomic Method With the Genotype × Environment Model (FGGE)

The model of Jarquín et al. (2014) including GE is described as

(5) $$y=\mu 1_{n}+e+g+\mathrm{ge}+\varepsilon$$

In this case, the response ***y*** is a column vector of size *n*×1 comprising observations from *k* environments, that is, ***y*** = (***y***_*n1*_,…,***y***_*ni*_,…,***y***_*nK*_)′, where ***y***_*n*_*i*__ denotes the vector of observations of the *i*th environment, and *n*_*i*_ is the number of observations in the *i*th environment, with $n=\sum_{i=1}^{k}n_{i}$ the total number of observations in *k* environments. Also, μ is the overall mean, vector ***e*** is a random effects of the environments of size *n*×1 with a normal distribution $e∼N\left(0,\sigma_{e}^{2}Z^{e}{\mathrm{EZ}}^{e^{\prime }}\right),$ where ***E*** could be an identity matrix of order *k*×*k* (where *k* represents the number of environments) or a variance-covariance matrix when some lines are repeated in some environments. Matrix ***Z***^*e*^ is the incidence matrix of size *n*×*k* that relates the ***y*** observations with the environments. Vector ***g*** denotes the genetic random main effects of size *n*×1 with normal distribution $g∼N\left(0,\sigma_{g}^{2}G\right)$, where ***G*** is a matrix of order *n*×*n*, which is usually computed as ***Z***^*g*^***KZ***^*g*′^, where ***Z***^*g*^ is an incidence matrix that relates the genotypes to the observations and ***K*** is the genomic similarity kernel matrix of lines. Vector ***ge*** represents the random effect of the genotype × environment interaction of size *n*×1 with a normal distribution $\mathrm{ge}∼N\left(0,\sigma_{ge}^{2}\mathrm{GE}\right)$, where ***GE*** is a known matrix of order *n*×*n*. Note that matrix ***GE*** can be constructed as ***G***#**Z**^*e*^***EZ***^*e*′^ where # represents the Hadamard product. The vector of random errors with homogeneous variance is normal $\varepsilon ∼N\left(0,\sigma_{\varepsilon }^{2}I_{n}\right)$.

### Genomic Approximate Kernel Methods With a Genotype × Environment Model (APGE)

We will focus on the main effects of the genotypes and the interaction effects to take advantage of the properties of the approximate kernel. Therefore, the approximate method is similar to the case of a single environment, that is, $g∼N\left(0,\sigma_{g}^{2}Q^{g}\right)$, where $G\approx Q^{g}=G_{n,m}G_{m,m}^{-1}G_{n,m}^{\prime }$, whereas for the random interaction $\mathrm{ge}∼N\left(0,\sigma_{ge}^{2}Q^{ge}\right)$, where $\mathrm{GE}\approx Q^{ge}={\mathrm{GE}}_{n,m}{\mathrm{GE}}_{m,m}^{-1}{\mathrm{GE}}_{n,m}^{{}^{\prime }}$.

Similarly, for the approximate method for a single environment, we can decompose $G_{m,m}^{-1}$ and ${\mathrm{GE}}_{m,m}^{-1}$ in such a way that model (5) could be approximated as:

(6) $$y=\mu 1_{n}+e+P^{g}f+P^{ge}l+\varepsilon$$

where $P^{g}=G_{n,m}U_{m,m}^{g}S_{m,m}^{g^{-\frac{1}{2}}}$, $P^{ge}={\mathrm{GE}}_{n,m}\;U_{m,m}^{ge}S_{m,m}^{ge^{-\frac{1}{2}}}$, and vectors ***f***, ***l*** are of order *m*×1.

In summary, the suggested approximate method described above can be implemented with the following steps:

Step 1. Randomly select *m* lines from the training set, extracting the same number of lines for each environment.
Step 2. To construct matrices ***G***_*m*,*m*_ and ***G***_*n*,*m*_, one could proceed by ordering matrix **X** = (**X**_*n*_1_,*p*_,..,**X**_*n*_*i*_,*p*_,…,**X**_*n*_*k*_,*p*_)′, and constructing $G_{m,m}=\frac{X_{m,p}X_{m,p}^{\prime }}{p}$, $G_{n,m}=\frac{X_{n,p}X_{m,p}^{\prime }}{p}$. Another way to proceed is to use matrix ***K***, if available, and construct matrices $G_{m,m}=Z_{m,c}^{g}K_{c,c}Z_{m,c}^{g^{\prime }}$ and $G_{n,m}=Z_{n,c}^{g}K_{c,c}Z_{m,c}^{g^{\prime }}$, where *c* represents the number of lines without replicates.
Step 3. Construct matrices GEm,m=Gm,mZk,me#Ek,kZk,me,GEn,m=GEn,mZn,ke#Ek,kZk,me, **P**^*g*^, **P**^*ge*^.
Step 4. With the previous matrices, model (6) can be fitted and the required genomic-enabled predictions can be obtained.

## Experimental Data

To evaluate the performance of the different methods (FS and AP) and models (SE and GE) (FSSE, FSGE, ASE, and APGE), we used two sets of wheat data; the first data set (**data set 1**) is a large data set and the second is a small data set (**data set 2**).

### Data Set 1 – Large Data Set

This data set was used by Pérez-Rodríguez et al. (2020) and comprises 45,099 wheat lines and genotypes with 6978 GBS markers. From the total number of 45,099 wheat lines, 7671, 9021, 9501, 9821 and 9015 wheat lines were evaluated in years 2013–2014, 2014–2015, 2015–2016, 2016–2017 and 2017–2018, respectively. Thus, this data set has 5 environments that represent 5 different years, and the lines in different years are different.

### Data Set 2 – Small Data Set

This data set includes the wheat data sets used by Crossa et al. (2010), and comprises 599 wheat lines evaluated in four different environments and genotyped with 1279 SNP markers.

### Assessing Prediction Accuracy of the Full Genomic and the Genomic Approximate Kernel models for Single-Environment and for GE

To assess the performance of method-model combinations FGSE and APSE, we used models 3 and 4, respectively, and drew 20 random samples, with 80% of the observations used for training and 20% for testing in each sample. We used all the data and made predictions for single environments for both FGSE and APSE methods. However, for the AP method, we used 5 different sample sizes (*m*); for **data set 1**, *m* = 4000, *m* = 2000, *m* = 1000, *m* = 500, and *m* = 100. The analyses were performed in each case (FGSE and APSE) for five of the cycles included in this study (Table 1 and Figure 2). For **data set 2**, *m* = 264, *m* = 132, *m* = 74, *m* = 32, *m* = 15 (Table 2 and Figure 3). In addition, Tables 1 and 2 show the % of variation of matrix ***K*** that would be explained by taking the first *m* eigenvalues from the decomposition of ***K***, that is, $\varphi =100\times \sum_{i=1}^{m}s_{i}/\sum_{i=1}^{n}s_{i}$ (as a measure of the decay of the eigenvalues).

**TABLE 1:** Data set 1.

	**Sample size of the training** *m*					
**Cycle (*n* = total number of lines)**	**Model FGSE** *m* **= all φ = 100**	**Model APSE** *m* **= 4000 φ = 99.7**	**Model APSE** *m* **= 2000 φ = 98.6**	**Model APSE** *m* **= 1000 φ = 97.2**	**Model APSE** *m* **= 500 φ = 96.0**	**Model APSE** m **= 100 φ = 92.5**

**CORR**						
Cycle 2017_2018 (*n* = 9015)	0.575 (0.016)	0.575 (0.016)	0.570 (0.015)	0.557 (0.017)	0.534 (0.017)	0.464 (0.02)
Cycle 2016_2017 (*n* = 9821)	0.483 (0.011)	0.483 (0.011)	0.477 (0.015)	0.465 (0.013)	0.447 (0.011)	0.386 (0.012)
Cycle 2015_2016 (*n* = 9501)	0.533 (0.013)	0.533 (0.013)	0.522 (0.014)	0.508 (0.013)	0.483 (0.014)	0.402 (0.016)
Cycle 2014_2015 (*n* = 9021)	0.494 (0.017)	0.493 (0.012)	0.485 (0.020)	0.470 (0.016)	0.441 (0.018)	0.318 (0.021)
Cycle 2013_2014 (*n* = 7671)	0.572 (0.015)	0.572 (0.015)	0.567 (0.016)	0.549 (0.015)	0.515 (0.016)	0.366 (0.004)
**PMSE**						
Cycle 2017_2018 (*n* = 9015)	0.282 (0.009)	0.282 (0.009)	0.284 (0.008)	0.290 (0.009)	0.300 (0.008)	0.336 (0.01)
Cycle 2016_2017 (*n* = 9821)	0.369 (0.009)	0.369 (0.010)	0.364 (0.010)	0.377 (0.010)	0.385 (0.010)	0.410 (0.011)
Cycle 2015_2016 (*n* = 9501)	0.304 (0.010)	0.304 (0.010)	0.309 (0.013)	0.315 (0.010)	0.326 (0.010)	0.356 (0.012)
Cycle 2014_2015 (*n* = 9021)	0.309 (0.012)	0.309 (0.013)	0.311 (0.011)	0.319 (0.013)	0.329 (0.013)	0.368 (0.016)
Cycle 2013_2014 (*n* = 7671)	0.413 (0.011)	0.413 (0.013)	0.413 (0.014)	0.429 (0.012)	0.451 (0.012)	0.508 (0.011)
${\hat{\sigma }}_{\varepsilon }^{2}$						
Cycle 2017_2018 (*n* = 9015)	0.247 (0.003)	0.250 (0.003)	0.262 (0.002)	0.275 (0.002)	0.293 (0.003)	0.330 (0.004)
Cycle 2016_2017 (*n* = 9821)	0.317 (0.003)	0.323 (0.003)	0.337 (0.003)	0.350 (0.003)	0.365 (0.003)	0.400 (0.003)
Cycle 2015_2016 (*n* = 9501)	0.255 (0.003)	0.257 (0.003)	0.279 (0.003)	0.297 (0.003)	0.315 (0.004)	0.357 (0.005)
Cycle 2014_2015 (*n* = 9021)	0.259 (0.003)	0.266 (0.003)	0.280 (0.003)	0.298 (0.003)	0.315 (0.004)	0.366 (0.004)
Cycle 2013_2014 (*n* = 7671)	0.313 (0.004)	0.324 (0.005)	0.358 (0.005)	0.391 (0.006)	0.424 (0.006)	0.501 (0.006)
**TIME (in seconds)**						
Cycle 2017_2018 (*n* = 9015)	3931	1710	707	345	174	47
Cycle 2016_2017 (*n* = 9821)	4350	1765	768	356	176	48
Cycle 2015_2016 (*n* = 9501)	4200	1750	759	375	184	49
Cycle 2014_2015 (*n* = 9021)	3850	1310	695	330	165	51
Cycle 2013_2014 (*n* = 7671)	2800	1135	533	247	134	44

*Prediction of 20 random samples with 80% of observations in the training set and 20% in the testing set for single site models for the 5 cycles. The FGSE model with a training set of size m = all and the % of variation retained of matrix **K** (φ) as φ = 100, and APSE for m = 4000, m = 2000, m = 1000, m = 500, and m = 100 wheat lines and φ = 99.7, φ = 98.6, φ = 97.2, φ = 96.0, and φ = 92.5. Average correlation between predictive and observed values (CORR). Predictive Mean Squared Error (PMSE), and residual variance $\left({\hat{\sigma }}_{\varepsilon }^{2}\right)$. Computing average time required including the time for preparing matrix **K** plus the time required for 20,000 iterations using the software R package BGLR (standard deviations for CORR, PMSE, and ${\hat{\sigma }}_{\varepsilon }^{2}$ in parentheses).*

**TABLE 2:** Data set 2.

	**Sample size of** *m*					
**Environment**	**Model FGSE** *m* **= all φ = 100**	**Model APSE** *m* **= 264 φ = 98.6**	**Model APSE** *m* **= 132 φ = 95.8**	**Model APSE** *m* **= 72 φ = 95.6**	**Model APSE** *m* **= 36 φ = 88.8**	**Model APSE** *m* **= 15 φ = 82.2**

**CORR**						
E1	0.506 (0.046)	0.501 (0.047)	0.468 (0.063)	0.425 (0.073)	0.362 (0.060)	0.266 (0.088)
E2	0.471 (0.068)	0.461 (0.062)	0.439 (0.066)	0.407 (0.071)	0.374 (0.060)	0.283 (0.072)
E3	0.384 (0.046)	0.384 (0.047)	0.381 (0.059)	0.359 (0.053)	0.318 (0.064)	0.267 (0.068)
E4	0.448 (0.051)	0.439 (0.05)	0.420 (0.048)	0.398 (0.053)	0.359 (0.050)	0.302 (0.053)
**PMSE**						
E1	0.771 (0.074)	0.776 (0.047)	0.806 (0.075)	0.848 (0.085)	0.899 (0.086)	0.957 (0.088)
E2	0.751 (0.08)	0.761 (0.078)	0.782 (0.081)	0.809 (0.092)	0.834 (0.077)	0.891 (0.090)
E3	0.821 (0.085)	0.817 (0.082)	0.822 (0.098)	0.837 (0.087)	0.863 (0.087)	0.892 (0.090)
E4	0.802 (0.098)	0.811 (0.090)	0.827 (0.096)	0.844 (0.097)	0.873 (0.096)	0.912 (0.090)
${\hat{\sigma }}_{\varepsilon }^{2}$						
E1	0.523 (0.041)	0.572 (0.038)	0.656 (0.035)	0.733 (0.037)	0.819 (0.037)	0.890 (0.040)
E2	0.587 (0.039)	0.635 (0.041)	0.707 (0.036)	0.768 (0.037)	0.840 (0.041)	0.902 (0.046)
E3	0.602 (0.039)	0.691 (0.043)	0.768 (0.048)	0.823 (0.041)	0.877 (0.045)	0.930 (0.048)
E4	0.598 (0.046)	0.652 (0.044)	0.720 (0.040)	0.775 (0.041)	0.833 (0.038)	0.890 (0.044)
**TIME (in seconds)**						
TE1	17	13.7	11	10.9	9.25	8.6
E2	17	13.7	11	10.9	9.25	8.6
E3	17	13.7	11	10.9	9.25	8.6
E4	17	13.7	11	10.9	9.25	8.6

*Prediction of 20 random samples with 80% of observations in the training set and 20% in the testing set for single site models for the 5 cycles. The FGSE model with the training set of size m = all and the % of variation retained of matrix **K** (φ) as φ = 100, and APSE for m = 264, m = 132, m = 72, m = 36, and m = 100 wheat lines and φ = 98.6, φ = 95.8, φ = 95.6, φ = 88.8, and φ = 82.2. Average correlation between predictive and observed values (CORR). Predictive Mean Squared Error (PMSE), and residual variance $\left({\hat{\sigma }}_{\varepsilon }^{2}\right)$. Computing average time required including the time for preparing matrix **K** plus the time required for 20,000 iterations using the software R package BGLR (standard deviations for CORR, PMSE, and ${\hat{\sigma }}_{\varepsilon }^{2}$ in parentheses).*

**Data set 1** is used for fitting the GE models, FGGE and APGE, using training cycles 2013–2014, 2014–2015, 2015–2016, 2016–2017 to predict cycle 2017–2018. For the FGGE model, it was computationally not possible to fit such a model using a standard laptop (computer 1, laptop) since the size of the training set is a ***G*** matrix of order 45099 × 45099. Therefore, we used the results from Pérez-Rodríguez et al. (2020; Chapter 13, Table 13.4), who used the same training data to predict cycle 2017–1018. These authors achieved a genomic-enabled prediction accuracy of 0.4263 using only markers. The prediction of the same cycle (2017–2018) used the approximate APGE model with only 25% of the total training lines from each cycle for the *m*, that is, matrices ***K***_*m*,*m*_, ***K***_*n*,*m*_ are manageable matrices of order 9021 × 9021 and 45099 × 9021, respectively. Table 3 shows the genomic prediction of each cycle taking one or more of the previous cycles as training. For model APGE, we used 25% of the total training set for each cycle as the size of *m*. For fitting model FGGE, we used another computer facility (computer 2) because the laptop (computer 1) could not fit the models.

**TABLE 3:** The models FGGE and APGE considering the size of *m*, as 25% of the original training set.

Cycle	Training	CORR	PMSE	${\hat{\sigma }}_{\varepsilon }^{2}$	TIME (h)
**Data set 1 modelFGGE (using *computer 2*)**					
Cycle 2014_2015	Cycle 2013_2014	0.222	2.45	0.317	4.96
Cycle 2015_2016	Cycle 2013_2014	0.328	0.525	0.287	11.10
	Cycle 2014_2015				
Cycle 2016_2017	Cycle 2013_2014	0.328	0.480	0.275	23.72
	Cycle 2014_2015				
	Cycle 2015_2016				
Cycle 2017_2018	Cycle 2013_2014	0.426	NA	NA	NA
	Cycle 2014_2015				
	Cycle 2015_2016				
	Cycle 2016_2017				
**Data set 1 model APGE (using *computer 1*)**					
Cycle 2014_2015	Cycle 2013_2014	0.206	1.08	0.363	0.68
Cycle 2015_2016	Cycle 2013_2014	0.347	0.408	0.309	2.80
	Cycle 2014_2015				
Cycle 2016_2017	Cycle 2013_2014	0.321	0.517	0.29	5.08
	Cycle 2014_2015				
	Cycle 2015_2016				
Cycle 2017_2018	Cycle 2013_2014	0.427	0.618	0.301	8.38
	Cycle 2014_2015				
	Cycle 2015_2016				
	Cycle 2016_2017				

**Environment**	**Training**	**CORR**	**PMSE**	${\hat{\sigma }}_{\varepsilon }^{2}$	**TIME (s)**

**Data set 2 model FGGE (using *computer 1*)**					
E1	E2	-0.166	1.520	0.532	175
	E3				
	E4				
E2	E1	0.511	0.912	0.600	178
	E3				
	E4				
E3	E1	0.469	0.879	0.577	180
	E2				
	E4				
E4	E1	0.311	.940	0.570	187
	E2				
	E3				
**Data set 2 model APGE (using *computer 1*)**					
E1	E2	-0.188	1.54	0.607	70
	E3				
	E4				
E2	E1	0.491	0.942	0.71	72
	E3				
	E4				
E3	E1	0.445	0.887	0.70	73
	E2				
	E4				
E4	E1	0.281	0.960	0.651	82
	E2				
	E3				

*Average correlation between predictive and observed values (CORR), Predictive Mean Squared Error (PMSE), and residual variance $\left({\hat{\sigma }}_{\varepsilon }^{2}\right)$. Computing average time required including the time for preparing the matrices G, GE plus the time required for 20,000 iterations using the software R package BGLR.*

For the small data set, **data set 2** is predicted with the rest of the environments using the full genomic FGGE (model 5); the variance-covariance matrices are of order 2396 × 2396, a size that does not cause any computational problem. For APGE, *m* was 25% of the training set of each environment (representing a total of 450 wheat lines, that is, 150 lines in each of the three environments used for training). Table 4 shows the variance component estimates for model APGE in **data set 2**.

**TABLE 4:** Estimated variance components for model APGE for **data set 1** and **data set 2**.

Testing	Training	${\hat{\sigma }}_{\varepsilon }^{2}$	${\hat{\sigma }}_{g}^{2}$	${\hat{\sigma }}_{ge}^{2}$
**Data set 1**				
Cycle 2014–2015	Cycle 2013–2014	0.3624	0.4680	0.3300
Cycle 2015–2016	Cycle 2013–2014	0.3087	0.2638	0.3337
	Cycle 2014–2015			
Cycle 2016–2017	Cycle 2013–2014	0.2916	0.22705	0.2956
	Cycle 2014–2015			
	Cycle 2015–2016			
Cycle 2017–2018	Cycle 2013–2014	0.3019	0.1886	0.2962
	Cycle 2014–2015			
	Cycle 2015–2016			
	Cycle 2016–2017			
**Data set 2**				
E1	E1	0.6070	0.3953	0.5576
	E3			
	E4			
E2	E1	0.7102	0.3183	0.1120
	E3			
	E4			
E3	E1	0.7001	0.3053	0.1356
	E2			
	E4			
E4	E1	0.6510	0.2981	0.1985
	E2			
	E3			

**FIGURE 1:**
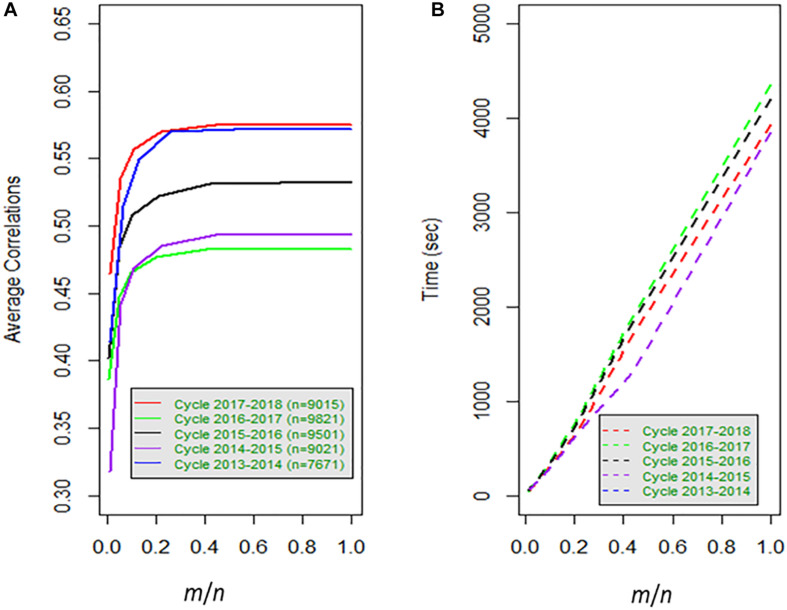
**(A)** Average correlation for 80% training and 20% testing for 20 random samples for data set1, versus the proportion of size *m* with respect to the total number of observations (lines) *n*; **(B)** time in seconds for each sample versus the proportion of size of *m* over the total number of lines (*n*).

**FIGURE 2:**
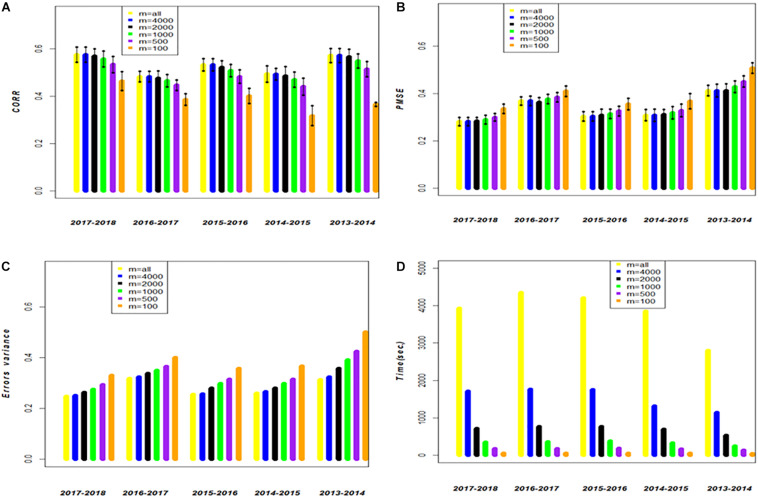
**Data set 1**. Models FGSE (yellow, *m* = all lines) and APSE (blue, black, green, purple and orange that correspond to *m* = 4000, *m* = 2000, *m* = 1000, *m* = 500, *m* = 100), **(A)** average correlation between observed and predictive values of FGSE and APSE models at different sizes of *m*; bars indicated 2 standard deviations) **(B)** average prediction mean squared error (PMSE) values of FGSE and APSE models at different sizes of *m*, **(C)** error variance of FGSE and APSE models (${\hat{\sigma }}_{\varepsilon }^{2}$) at different sizes of *m*, and **(D)** time in seconds to fit FGSE and APSE models at different sizes of *m*.

As criteria for all model-method combinations (FGSE, APSE, FGGE, APGE) used to evaluate the prediction accuracy and computing time, we employed: (1) the mean Pearson’s correlation between the predictive and the observed values (CORR), where the predictive values are extracted from the mode of the Bayesian predictive distribution; (2) the prediction mean squared error PMSE is the mean of the squared difference between the predictive and the observed value; (3) the fitted models with the residual error variance $\left({\hat{\sigma }}_{\varepsilon }^{2}\right)$; and (4) the time (TIME) for constructing the matrices and fitting the model (Tables 1–3 and Figures 1, 2). For model APGE, we estimated the variance components of the main effects $\sigma_{g}^{2},$ the interaction effects $\sigma_{ge}^{2}$ and random error $\sigma_{\varepsilon }^{2}$ (Table 4).

### Software

To fit the models we used Bayesian Ridge Regression from BGLR (de los Campos and Pérez-Rodríguez, 2018), because it is a free software that focuses on genomic predictions, and it is flexible, allowing users to fit complex models including multi-kernels, main effects and G × E effects. BGLR is very well documented with a large number of clearly explained examples that can be found in https://github.com/gdlc/BGLR-R.

Models were fitted and predictions were made using 20,000 iterations and discarding the first 3000 iterations and using a thinning of 2. Initially the Raftery and Lewis (1992) criteria was employed to determine the minimum of iterations, the “burn in” and the “thin.” also we made visual observations of graphs representing the Monte Carlo Markov Chain to make sure a good mixture was achieved.

### Hardware

***Computer 1*** is a laptop with a processor intel^®^ Core i5^TM^ i5-7300 HQ CPU@ 2.5 GHz 2.5 GHz, RAM 16 GB, Operative System of 64 bit, with processor x64.

***Computer 2,*** vendor_id : AuthenticAMD, cpu family : 16, model: 9, model name;: AMD Opteron(tm) Processor 6140, stepping : 1, microcode : 0x10000c4, cpu MHz : 2600.185, cache size : 512 KB.

### Data Repository

The 5 phenotypic and genotypic data sets (cycle 13–14, cycle 14–15, cycle 15–16, cycle 16–17, and cycle 17–18) comprising **data set 1** can be downloaded from the following link: http://hdl.handle.net/11529/10548425. As already mentioned, **data set 2** can be found in Crossa et al. (2010), or as an illustrative example in the BGLR R package (de los Campos and Pérez-Rodríguez, 2018) or in a large number of other genomic-based studies that have used this experimental data set.

## Results

### Results of FGSE and APSE for Large (Data Set 1) and Small (Data Set 2) Data

For large **data set 1**, Table 1 and Figure 2 show the prediction accuracy of 20 random cross-validation partitions, where in each sample, 20% of the wheat lines are predicted from a training set of 80% of the total wheat lines for the 5 cycles. The first column contains the results of the FGSE (model 3) using all wheat lines in each cycle (*m* = all). It shows the average correlation (CORR) of the 20 random samples of 20% of the wheat lines in the testing set, as well as the mean of the 20 PMSEs and the mean of the 20 estimations of the residuals (${\hat{\sigma }}_{\varepsilon }^{2}$). Finally, it shows the TIME invested in each sample of the training-testing combination for 20,000 iterations. Columns 2–6 in Table 1 provide the results of CORR, PMSE, ${\hat{\sigma }}_{\varepsilon }^{2}$, and TIME for *m* = 4000, *m* = 2000, *m* = 1000, *m* = 500, *m* = 100 wheat lines of APSE (model 4), randomly selected in order to compute ***K***_*m*,*m*_, and ***K***_*n*,*m*_.

The behavior of the cycles is similar for FGSE and APSE for 4000 wheat lines for ***K***_*m*,*m*_, ***K***_*n*,*m*_, but genomic-enabled prediction values are lost as the number of lines included in the training set is reduced; this is reflected in the decrease of the CORR, and the increase in PMSE and (${\hat{\sigma }}_{\varepsilon }^{2}$). For example, for cycle 2017_2018, FGSE with all observations had a CORR of 0.575, a PMSE of 0.282, and an estimated ${\hat{\sigma }}_{\varepsilon }^{2}$ of 0.247. Interestingly, these results are similar to those found for the APSE when only 4000 wheat lines were used as training (55% of the total original training set), with a CORR of 0.575, a PMSE of 0.282 and an estimated ${\hat{\sigma }}_{\varepsilon }^{2}$ of 0.250. Furthermore, when APSE used only 2000 wheat lines as training (28% of the total original training set), the genomic-enabled prediction accuracy slightly decreased to a CORR of 0.570, and the PMSE had a small increase with PMSE = 0.254 as a result of a less fitted value ${\hat{\sigma }}_{\varepsilon }^{2}=0.262$ (Table 1).

The genomic-enabled prediction decreases for smaller sample sizes (*m*) of 1000, 500, and 100, where CORR takes values of 0.557, 0.534, and 0.46, respectively, increasing PMSE to 0.290, 0.300, and 0.336, as well as the estimated ${\hat{\sigma }}_{\varepsilon }^{2}$ values to 0.275, 0.293, and 0.330, respectively. The computing TIME decreases almost linearly (3931, 1710, 707, 345, 174, 47 seconds) for the decreasing sample size (*m*). The results of the different sample sizes of *m* and the correlations from Table 1 (**data set 1**) are also displayed in Figure 1A where, for example, for cycle 2017–2018 for *m*/*n* = 0.22, the average correlation for the genomic-enabled prediction is 0.570, whereas for *m*/*n* = 1.0, the average correlation is 0.575. It is interesting to observe that the computational time required decreases linearly as the size of *m* decreases in relation to the size of *n* (Figure 1B).

**FIGURE 3:**
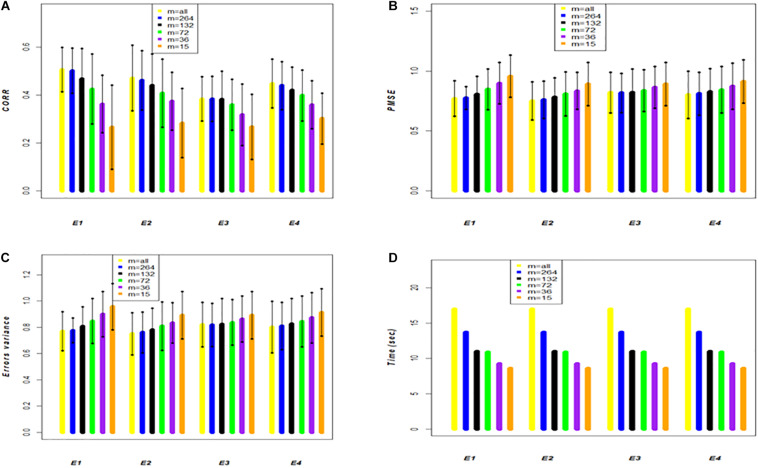
**Data set 2**. Models FGSE (yellow, *m* = all lines) and APSE (blue, black, green, purple and orange that correspond to *m* = 4000, *m* = 2000, *m* = 1000, *m* = 500, *m* = 100), **(A)** average correlation between observed and predictive values of FGSE and APSE models at different sizes of *m*; bars indicated 2 standard deviations) **(B)** average prediction mean squared error (PMSE) values of FGSE and APSE models at different sizes of *m*, **(C)** error variance of FGSE and APSE models (${\hat{\sigma }}_{\varepsilon }^{2}$) at different sizes of *m*, and **(D)** time in seconds to fit FGSE and APSE models at different sizes of *m*.

The results of the small **data set 2** shown in Table 2 and Figure 3 have the same structure as those shown in Table 1 and Figure 2 for **data set 1**; however, the results are different. These data have 599 wheat lines all evaluated in 4 environments (E1, E2, E3, and E4) (Crossa et al., 2010). Each of the 20 random samples with 479 wheat lines in the training set and 120 lines in the testing set had varying results; however, compared to the results obtained with the large data set (**data set 1**), these results are quite different. The first column shows the results of the full genomic model (FGSE model 3) using all the data, and when compared with the APSE (model 4) with *m* = 264 (55% of the total training population), the CORR decreased slightly in 3 of the 4 environments; for example, in E1 it decreased from 0.506 to 0.501, whereas in E2 went from 0.47 to 0.461, it stayed the same in E3 and decreased in E4 from 0.448 to 0.439. Similar patterns were found for PMSE and ${\hat{\sigma }}_{\varepsilon }^{2}$.

When *m* = 132 lines (28% of the total original size of the training population), the decrease in CORR was severe in E1 and E2, decreasing to 0.468 and 0.439, respectively, but less so in E3, where it decreased to 0.381 as a consequence of a decrease in the fit with ${\hat{\sigma }}_{\varepsilon }^{2}$ of 0.656, 0.707, 0.766 and 0.720, respectively, in E1, E2, E3 and E4. The decreasing trend in CORR increased as *m* decreased; for example, in E1, when *m* = 74, or 36 or 15, CORR was 0.425, 0.362 and 0.262, respectively, and ${\hat{\sigma }}_{\varepsilon }^{2}$ increased to 0.730, 0.819 and 0.890. However, in contrast to data set 1, the mean computing time (TIME) for each of the 20 samples of the random cross-validation did not decrease in the same proportion as those due to the size of the sample.

Tables 1 and 2 and Figure 1 indicate that the differences in genomic prediction with respect to the full models depend more on the size of *m*, that is, the larger the *m*, the smaller the differences with the full model (*m* = all). Another important indicator is φ, because when φ > 98, the genomic-enabled prediction accuracy of the approximate model is equal to that of the full models; when φ < 98, the results of the approximate models are less precise than those obtained from the full model.

### Results of FGGE and APGE for Large (Data Set 1) and Small (Data Set 2) Data

Table 3 shows the genomic-enabled prediction accuracy for models FGGE y APGE for the two groups of data. To predict cycle 2017–2018 from **data set 1** using the previous 4 cycles with the full genomic GE model (FGGE, model 5), it is necessary to manipulate two large covariance matrices, one for the main effects of the genomic (G) model and another matrix for the interaction (GE) of order 45099 × 45099. It was not possible to manage this matrix size with the current conventional laptop (computer 1) used to analyze these data; therefore, we used the genomic-enabled prediction accuracy recently reported by Pérez-Rodríguez et al. (2020) as a reference. The authors used and reported a genomic prediction accuracy of 0.426 for cycle 2017–2018 using all the other cycles as a training set.

Using the approximate model APGE (model 6) and only 25% of the total training set, that is, *m* = 9021, such that matrices ***K***_*m*,*m*_, and ***K***_*n*,*m*_, are now of manageable sizes of order 9021 × 9021 and 45099 × 9021, respectively, this gives a genomic prediction accuracy of 0.427, with a residual variance of 0.302, that is, there is no loss of genomic prediction accuracy with respect to the full genomic models with GE (FGGE model 5). The computing time required, including the time for preparing the matrices for the approximation method, and the time for the eigenvalue decomposition and the 20,000 iterations, was 30,670 seconds. This was very similar for the prediction of the other cycles, and the only differences were in the computing time consumed between FGGE and APGE; this difference exponentially increased with the total number of training data.

When we used **data set 2** to predict environment E4 using environments E1, E2, and E3 as training and using FGGE, the required covariance matrices were of order 2396 × 2396, which does not pose any problems for their storage and manipulation. The prediction accuracy achieved by the FGGE for the genomic-enabled prediction of E4 was 0.311, with a PMSE of 0.94, a residual variance ${\hat{\sigma }}_{\varepsilon }^{2}$ of 0.57, and a duration time of 187 seconds. When using the approximate model APGE (model 6), we selected 25% of the training set (480 wheat lines) and found a decrease in the genomic prediction accuracy of 0.281 compared to the FGGE, an increase in the PMSE of 0.960, and an increase in the residual variance with respect to model FGGE of ${\hat{\sigma }}_{\varepsilon }^{2}$ = 0.651, with a faster computing time (82 seconds) than model FGGE. When predicting the other environments, the results were similar regarding the differences in the correlations between models FGGE and APGE.

Table 4 shows the estimated variance components for model APGE. It can be observed that for **data set 1**, the variance components for the main effects and the interactions were of similar magnitude, indicating the importance of both types of effects. For **data set 2**, the interaction variance component is relatively smaller than the main effects.

## Discussion

The main objective of this study was to show that the approximate kernel method offers a good solution for the large data sets usually encountered in genomic-enabled prediction when Bayesian linear mixed models need to be fitted. The usual problem in genomic prediction is that the number of markers (covariates, *p*) is much larger than the number of observations (*n*). However, the number of observations is also large, so performing matrix decomposition requires very intense computing in terms of time, storage capacity, etc. Approximate kernels allow matrix manipulation and storage, thus saving storage resources and computing processing time. In some cases, genomic prediction accuracy does not decrease much, but in other cases, the loss of precision is indeed important. This depends mainly on the size of *m* and on how fast the decrease in the eigenvalue decomposition of kernel ***K*** occurred. A rapid decrease in the eigenvalues indicates that with only a few singular values, a high percentage of the important variation could be retained. The variance retained using φ (Pocrnic et al., 2016) indicated the percentage of variation retained for a certain number of eigenvalues.

**Data sets 1** and **2** were fitted using the full genomic (FG) method and the approximate model (AP) for the single-environment model with certain percentages of the points selected from the total training set similar in the two data sets (55, 28, 14, 7, and 2% of the total training set). The size of *m* influenced the precision of the predictions. In **data set 1**, the genomic prediction accuracy was higher at 55 and 28% and slowly declined as the size of *m* decreased; this decrease in the prediction accuracy was smoother in **data set 1** than in **data set 2.** One of the reasons for these differences in prediction accuracy between the two data sets could be due to the rank of kernel ***K***. For example, in **data set 1** for cycle 2017–2018, kernel ***K*** (of order 9015 × 9015) had a rank of 7017, whereas for **data set 2**, the rank of matrix ***K*** was 598; that is, **data set 1** had more degrees of freedom than **data set 2**. A common feature of both data sets is the rapid decline in the singular values of their kernels; this is measured by φ as the percentage of variance retained by ***K*** using a certain number of singular values (size of *m*). The empirical results suggested using φ > 98 to avoid losing precision. This result is in agreement with that suggested by Misztal (2016). This could be used as a rule of thumb to select the minimum size of *m* that would return a φ > 98.

The rapid decline in the singular value of kernel ***K*** favors the use of the approximate kernel ***Q***, as suggested by Wang et al. (2015). Therefore, the rapid decline in the singular value of kernel ***K*** also favors the use of other methods that improve the computer speed, such as principal component regression using the original matrix ***K***. However, if the data are large, intense computational efforts are required to construct matrix ***K***, with an exponential requirement of computing capacity for eigenvalue decomposition. On the other hand, the approximate method requires a matrix of much lower order. When using an *m* associated with φ > 98, we do not expect significant differences in the prediction accuracy of the approximate model and the full model; also, no differences between the approximate model and the principal component regression model are expected using a similar size of *m*; however, when φ≪98, more differences are expected between the approximate model and the full genomic models but less with the principal component regression model.

In relation with the necessary computing time, the AP method applied to **data set 1** showed that the saving of computing time increases when the size of *m* decreases, whereas for **data set 2**, this also occurs but in different proportions because the data are of much lower dimension than those in **data set 1**. In general, the results of this study indicated that the computing time used to fit the full model increases exponentially with the number of observations *n*; this also applies to the approximate models. These results are in agreement with those of Wang et al. (2015), who commented that “most kernel-based methods have a computational complexity of order *O*(n^3^). This is prohibitive when we have large-scale training samples. The low-rank spectral reconstruction of a kernel can be performed by the Nyström method, which can speed up many regression-oriented algorithms. The approximation quality of these methods is protected by a reasonable and key assumption that the genomic data, like most other large data, live in a lower dimension space and the spectra of the kernel matrices often decay quickly.”

Figure 2A (**data set 1**) and Figure 3A (**data set 2**) display the predictions of the different years (cycles) for the FGSE (yellow, *m* = all lines) and APSE (blue *m* = 4000 lines; black *m* = 2000 lines; green, *m* = 1000 lines; purple, *m* = 500 lines and orange *m* = 100 lines). The pattern of the predictions are kept similar (2 times the standard deviations) for both models, FGSE and APSE, but changing the average correlations based on the size of *m* indicating congruence among the predictions of the 20 samples of different sizes of *m* that were randomly selected to form the training set. Also, it can be observed that for FGSE and APSE models, the sizes of *m* (yellow, blue and black, *m* = all lines *m* = 4000 lines and black *m* = 2000 lines, respectively) did not change the prediction accuracy of the unobserved wheat lines in the testing set much. In addition, note that in Figures 2C, 3C, the residual variance increased as the size of *m* decreased, indicating that the AP model does not produce overfitting.

It is indeed in the GE models where approximate kernels could have the greatest utility because the covariance matrix (G) of the main effects of markers and the GE are, in general, large matrices and the fit of the models is very slow computationally. The fit of model APGE for **data set 1**(Table 3) did not lose prediction accuracy when fitted with approximate kernels of lower rank as compared with the ones required by the FGGE, with an important reduction in the computing time. The APGE reduced the time required to prepare the matrices and to fit the model with 20,000 iterations to 8.5 h, when it takes days on a big server. For data set 2, the results were not that good; nevertheless, the precision did not decrease much, but the reduction in time was important.

Table 4 shows the variance components of the two data sets for model APGE. The magnitude of the variance components shows that the model captured the main effects as well as the interactions. Although φ is a good indicator for explaining the relationship between the decay of the singular values, unfortunately it is not always possible to estimate the decrease in the prediction accuracy and the adequate size of *m*.

Using the approximate kernel of this study, authors like Seeger et al. (2003); Snelson and Ghahramani (2006) and Titsias (2009) show examples with large numbers of observations (*n*), while the covariates (*p*) are continuous and of low dimensions. The *n*≫*p* implies the existence of redundant information (more degrees of freedom available); this allows using approximate kernels or a sparse Gaussian process (Rasmussen and Williams, 2006) in a very efficient manner. All these propositions emphasize the size of *m*, but also indicate which observations to choose. To deal with the selection of observations, some authors propose selecting those that minimize the trace of the matrix differences between the original matrix ***K*** and the approximate matrix ***Q*** (Rasmussen and Williams, 2006). Other authors propose maximizing the marginal likelihood based on the variational inference (Titsias, 2009; Hensman et al., 2013), where *m* observations are considered hyper-parameters. Nevertheless, for the linear mixed models used in genomic prediction, the high number of covariates (markers) may require investing important additional computing time for selecting the observations comprising *m*. On the other hand, empirical results show that selecting the observations at random (Tables 1–3) works all right because the main constraint is the size of *m*. These results are in line with the approximate kernel developed in animal breeding by means of pedigree and genomic selection for determining the breeding values performance of large numbers of animals (Misztal, 2016). However, in plant breeding, methods for efficiently selecting the observations comprising *m* need to be studied further, probably by selecting *m* lines using population substructure and diversity criteria such as the ones proposed by Akdemir (2014); Jeong et al. (2017).

## Conclusion

The approximate kernel methods used in this study are very promising because they allow a significant reduction in computing time and data manipulation of large data sets, without significant loss of prediction performance.

Results of model APSE for **data set 1** show a good performance on the genomic-enabled prediction accuracy compared with the full models with APSE employing an important decrease in computing time with respect to the full model. This can be explained by the rapid decrease in the singular values and their ability to capture important information, since with only 25% of the singular values, 98% of the total information was retained. For **data set 2**, model APSE does not have the same prediction performance as for **data set 1.** On average, genomic-enabled prediction accuracies decreased rapidly when the size of *m* decreased; however, the variability of the predictions was maintained with respect to the full model. In **data set 2**, the decay of the singular values was less rapid than that observed for **data set 1**, that is, 25% of the singular values retained 95% of the information.

For the very large **data set 1**, the results of model APGE with the size of *m* representing only 25% of the total number of lines gave an excellent correlation between predictive and observed values, along with an important saving of computing time. For the small **data set 2**, the APGE model gave better results than model APSE, and the decrease in the correlation was less compared to that of the full model when 25% of the total lines were used in *m*. In both data sets, the APGE model with fairly large G × E interactions of the variance components indicates that this variability will indeed increase the genomic-enabled prediction accuracy with respect to models that only include the main effects.

We also observed that the larger the data sets are, the more benefits can be obtained from the approximate kernel methods. However, for their successful implementation, two important factors should be taken into account: (a) the number of lines (*m*) that need to be used for approximating the kernel, and (b) the amount of information that can be retained in the approximate kernel (φ). According to our empirical study, we observed that for large data sets, a φ > 98% and a size of *m*> 50% of the total training observations are required for single-environment analyses, and *m* > 25% for GE analyses prevent important decreases in genomic-enabled prediction accuracy while obtaining time savings in computing resources.

Results of this study indicated that the proposed approximation could be an alternative to genomic prediction when the number of observations is large and the construction and storage of the large kernel matrices is difficult and it takes excessive computing time to fit models FGSE and FGGE. Regarding φ, although it is a good indicator of the variance retained by the singular values and thus for determining the adequate size of *m*, unfortunately, in practice it is not possible to compute it. Therefore, further research on this subject is needed for selecting the size of *m*. However, the results obtained are promising because they provide a partial solution to an important problem of genome-based prediction models.

## Data Availability Statement

The 5 phenotypic and genotypic data sets (cycle 13–14, cycle 14–15, cycle 15–16, cycle 16–17, and cycle 17–18) comprising data set 1 can be downloaded from the following link: http://hdl.handle.net/11529/10548425. As already mentioned, data set 2 can be found in Crossa et al. (2010), or as an illustrative example in the BGLR R package (de los Campos and Pérez-Rodríguez, 2018) or in a large number of other genomic based studies that have used this experimental data set.

## Author Contributions

JCu developed the idea, ran the analyses, and wrote the article. JCr discussed the original idea, wrote the manuscript, and helped JCu to present the new idea in several data set from CIMMYT. JM discussed the original ideas, and contributed with new insights when writing the article. PP-R contributed with the computing codes for running the software. OM-L contributed to reading and completing the models. ML read the manuscript and edited parts of the several drafts. All authors contributed to the article and approved the submitted version.

## Conflict of Interest

The authors declare that the research was conducted in the absence of any commercial or financial relationships that could be construed as a potential conflict of interest.
